# Antenatal anaesthesia clinics – the way forward?

**DOI:** 10.1186/s13584-018-0244-x

**Published:** 2018-08-21

**Authors:** Felicity Plaat, Catherine Harris

**Affiliations:** Queen Charlotte’s Hospital, London, UK

## Abstract

Specialist antenatal clinics are increasingly being used to enable anaesthetists to evaluate pregnant women with co-morbidities and those at high risk of obstetric complications. In this journal a team from Israel describe the process of setting up and running such a clinic over a 14 year period. One of the challenges they identify was the limited referral of high risk women. Based on UK and US literature, the use of structured referral tools, clear criteria for referral and regular antenatal multidisciplinary meetings may help to address this.

## Main text

For several years antenatal anaesthesia clinics have been part of the management of high risk parturients around the world. In the paper by Weiniger et al [1] the authors discuss the development of their clinic in Israel over the past 14 years. They start with the premise that medical co-morbidity is becoming a major cause of maternal mortality and the care of pregnant women with concurrent disease can be improved by cohesive, multidisciplinary care, including planned anaesthesia. The medical conditions of the women that attend their anaesthesia clinic is described in detail. Methods of referral, organizational aspects of the clinic and clinical outcomes are explored through a selection of case vignettes. The limitations to and challenges in providing an antenatal anaesthetic assessment service throughout Israel are discussed.

The reasons for referral are similar to those found in the UK, with one big difference. In the Israeli study the majority of women were referred for musculoskeletal conditions. Although this used to be the case in both the UK [2] and Canada [3], recently this has been superseded by obesity. In the past 10 years this was found to be the most common reason for referral in a host of studies [4–7]. Musculoskeletal conditions remain the second most common reason for referral. This begs the question why obese parturients are not being seen antenatally in Israel? The evidence does not suggest that it is because few Israeli parturients are obese: in fact a recent report put the proportion of Israeli women who were obese in 2015 at 24% [8]. Both the British Royal College [9] and the American College of Obstetrics and Gynaecology [10] recommend antenatal anaesthesia assessment of these women.

Another notable difference revealed in the article is in the overall numbers seen in the anaesthetic clinic compared to the UK experience. Over the 14-year period, 451 parturients attended the clinic; an average of 32 women per year [1]. These numbers are low in comparison to a single London teaching hospital where 300 women are seen each year [5]. The authors estimate that they should see up to 7000 pregnant women with cardiac disease alone over the 14 year period but they were only referred 66. From the information given it is difficult to assess the overall numbers that should be expected. One of the criticisms of this paper is the lack of denominator data which means that many assumptions are based on data from other populations: for example the authors’ estimates of morbidity are based on Brazilian data [11]. The prevalence of medical co-morbidities among Israeli parturients would illuminate the data provided.

Analysis of the numbers does seem to confirm that only a small proportion of women who are eligible to be seen actually attend the clinic. The reason is likely to be multifactorial. A of lack of awareness of the clinic and the need for anaesthetic antenatal assessment of high-risk women, the lack of well defined criteria for referral and/or a well structured referral process are implicated. It is also the case that implementation of a new service takes time, requires regular review and audit to grow and become established.

A survey of practice published in 2005 demonstrated that only 30% of UK obstetric units ran a formal antenatal anaesthetic clinic [12]. A repeat survey 4 years later showed that “*a significant improvement in the prevision of antenatal clinics which are now ran in 70% of units*” [13]. This change may have at least in part have been driven by national guidelines produced by various anaesthetic societies. Guidance published in 1998 stated that there should be early anaesthetic involvement in high-risk patients and tertiary referral centres should consider providing an antenatal anaesthetic clinic [14]. By 2013 the guidance had become more specific: there should be adequate consultant sessions to cover anaesthetic clinics and “*anaesthetists should encourage and facilitate consultation in the antepartum period by making themselves available when antenatal clinics are in progress and by ensuring clear lines of referral. A system for the antenatal assessment of high-risk mothers should be in place with 24-hour access to the information on the delivery suite* [15]”. Recommendations from reports on maternal morbidity and mortality are also likely to have driven this increase in anaesthetic antenatal clinic provision. Poor communication between disciplines, sub-standard care and lack of effective antenatal planning of high risk women has been repeatedly highlighted. Those women that should have an antenatal anaesthetic assessment are enumerated. The Royal College of Anaesthetists in the UK makes allotting time for antenatal assessment one condition for accreditation of anaesthetic units [16]. There is similar guidance from Royal College of Obstetrics and Gynaecology [9, 17]. Making anaesthetic antenatal assessment facilities a requirement or at least strongly recommended by national authorities will likely to increase uptake.

In Israel criteria for referral for antenatal anaesthetic assessment were set by the Ministry of Health in 2011 [1]. These do not include some of the most common conditions encountered by Weiniger et al. such as musculoskeletal conditions, anaesthetic issues and various neurological conditions. Butwick [18] has emphasised that criteria for referral may need to be adapted to the spectrum of diseases in a unit’s catchment area. Clear criteria make referral easier and avoids overburdening clinics. Use of a formalised checklist agreed by a multidisciplinary panel including obstetric anaesthetists, is widely endorsed [18–20]. Such checklists, completed at the first antenatal visit, are widely used in the UK. Clarity is needed as to who can make referrals [19]. In the UK both midwives and obstetricians can do so.

The impression given of the referral process in Hadassah Medical Centre is that it is ad hoc: family and gynaecological physicians are mailed with details of the clinic and in Health Management Organisation clinics there are notices advising high-risk pregnant women to make an appointment. The impact of such measures, such as the level of awareness of the service by potential referring physicians, is unknown. In the UK, the increase in number of antenatal anaesthetic clinics occurred concurrently with an increase in the use of formal referral processes as described above [21]. In the USA Butwick [18] found that formalising the anaesthetic assessment via implementation of a regular clinic, improved the quality and quantity of referral by local obstetricians.

Despite increasing number of high risk women being seen by anaesthetists antenatally, evidence that this improves maternal outcome is lacking [18]. In the UK in 2005 a significant minority of obstetric anaesthetists could not see any advantage in instituting a formal clinic process [12]. Five years later 70% of units had a clinic but nearly ¼ of anaesthetists surveyed felt that the demand meant the services provided were not adequate for the need [13]. This growing enthusiasm may be a response to the growing number of high risk cases, as well as the increase in women that require anaesthetic interventions during their perinatal period [22]. Evidence from the UK and other European countries demonstrate much mortality and serious morbidity is secondary to substandard care, mismanagement and inadequate early clinical risk assessment [22–25]. Early anaesthetic assessment allows investigations to be undertaken, referrals made and care plans formulated. The logical conclusion must be that identification of high risk women followed by careful decision making, by senior clinicians in the formal environment of an antenatal anaesthetic clinic may reduced mortality and morbidity.

As Weiniger et al [1] points out the impressively low rates of maternal mortality in Israel may act as a disincentive to developing anaesthetic clinics. However serious morbidity data might paint a different picture. A recent report on mortality in UK [22] showed 75% of the women who died had pre-existing medical conditions, the majority of which were present antenatally. Deaths due to concurrent medical (and psychiatric) causes (so- called ‘indirect deaths) were more common than deaths due to obstetric complications, (‘direct deaths’) A review of pre-existing morbidities in the childbearing population in Israel could help promote awareness of the need for antenatal anaesthetic assessments.

As well as ensuring that allied specialties are aware of the services that each other are providing, a regular meeting of the multidisciplinary team allows each specialty to share knowledge and appreciate the concerns of other disciplines. In our experience, a weekly meeting of this nature regularly and reliably generates referral to the anaesthetic clinic. In the UK national guidance states that “there should be multidisciplinary clinical governance structures in place to enable the oversight of all birth settings” [26]. Weiniger et al describe how they plan to run regular cardiac multidisciplinary clinic, which hopefully will have the same effect.

## Conclusions

Weiniger et al. are to be applauded for this initiative, which has the potential improve maternal safety. They discuss the challenges with honesty and clarity. Regular review and audit of clinic activity and management of high risk women should provide robust data to promote their service both to those who refer and also to the relevant authorities who can produce guidance at a higher level. Through gradual improvement to the structure and organisation, the delivery of anaesthetic antenatal assessment in Israel is likely to grow exponentially.

## References

[1] Weiniger CF, Einav S, Elchalal U (2018). Concurrent medical conditions among pregnant women – ignore at their peril: report from an antenatal anaesthesia clinic. Israel J Health Policy Res.

[2] Cormack C, Francis S, Yentis S (2007). Antenatal anaesthetic assessment of the pregnant women. Anaesth Intensive Care Med.

[3] Rosaeg OP, Yarnell RW, Lindsay MP (1993). The obstetrical anaesthesia assessment clinic: a review of six years experience. Can J Anaesth.

[4] Salaunkey KMP, Radhakrishnan D, Mannakkara C (2007). An overview of a high-risk obstetric anaesthetic clinic. Reg Anaesth Pain Med.

[5] Wendler R, Schroeder F, Hammond S. Department of anaesthesia / maternal medicine annual report. St Georges Hospital. 2014. http://www.stgeorges.nhs.uk/ObstetricAnaesthetic Clinic_2014_Annual report. Accessed 16 July 2018.

[6] Tam E, Nadella V, Puttaswamy R, Williams M (2018). Role of the antenatal anaesthetic assessment clinic in the management of high-risk parturients in a district general hospital. Int J Obstet Anaesth.

[7] Uwubamwen NA, Eloft E (2011). Out-patient obstetric anaesthesia clinic: a one-year review of antenatal assessment of high-risk obstetric patients. Int J Obstet Anaesth.

[8] The GBD 2015 Obesity Collaborators (2017). Health effects of overweight and obesity in 195 countries over 25 years. N Engl J Med.

[9] Modder J, Fitzsimons KJ. Management of women with obesity in pregnancy. Centre for maternal and child enquiries / Royal College of obstetricians and gynaecologists joint guideline. 2010. https://www.rcog.org.uk/en/guidelines-research-services/guidelines/management-of-women-with-obesity-in-pregnancy. Accessed 16 July 2018.

[10] Riley LE, Stark AR (2012). Guidelines for perinatal care. TheAmerican Academy of pediatrics and American College of obstetrics and gynecology- 7th edition.

[11] Cecatti JG, Costa ML, Haddad SM (2015). Network for surveillance of severe maternal morbidity: a powerful national collaboration generating data on maternal health outcome and care. BJOG.

[12] Rai MR, Lua SH, Popat M, Russell R (2005). Antenatal assessment of high-risk pregnancy: a survey of UK practice. Int J Obstet Anaesth.

[13] Kelkar A, Darbar A, Brooks H (2010). Antenatal anaesthetic assessment of the high risk parturient: an OAA approved survey of UK maternity units. Int J Obstet Anaesth.

[14] Thomas TA, Baird WLM, Balance JHW (1998). Guidelines for obstetric Anaesthesia services.

[15] Plaat F, Bogod D, Bythell V, et al. OAA / AAGBI Guidelines for obstetric anaesthetic services 2013. London: Association of Anaesthetists of Great Britain & Ireland Obstetrics Anaesthetists’ Association; 2013. https://www.aagbi.org/sites/default/files/obstetric_anaesthetic_services_2013.pdf. Accessed 15 July 2018.

[16] Anaesthesia Clinical Services Accreditation Standards. Royal College of Anaesthetists 2018. http://rcoa.ac.uk/ACSA

[17] Pavord S, Rayment R, Madan B (2017). Management of inherited bleeding disorders in pregnancy. Green-top guideline no. 71. BJOG.

[18] Butwick AJ, Carvalho B (2007). Can we improve maternal outcome for high-risk obstetric patients?. Int J Obstet Anaesth.

[19] Nosakhare H, Uwubamwen NA, Verma D (2016). Antenatal anaesthetic assessment of obstetric patients. Anaesth Intensive Care Med.

[20] Zuokumor P (2013). Antenatal anaesthetic assessment of the obstetric patient. Anaesth Intensive Care Med.

[21] Sivaprakasam J, Purva M, Russell IF (2008). Identifying high-risk obstetric anaesthetic patients: a survey of UK practice. Int J Obstet Anaesth.

[22] Knight M, Kenyon S, Brocklehurst P, Neilson J, Shakespeare J, Kurinczuk JJ, on behalf of MBRRACE-UK (2014). ‘Saving lives, improving mothers’ care - lessons learned to inform future maternity care from the UK and Ireland confidential enquiries into maternal deaths and morbidity 2009–12.

[23] Bowyer L (2008). The Confidential Enquiry into Maternal and Child Health (CEMACH). Saving Mothers’ Lives: reviewing maternal deaths to make motherhood safer 2003–2005. The Seventh Report of the Confidential Enquiries into Maternal Deaths in the UK. Obstet Med.

[24] Lewis G (2004). The confidential enquiry into maternal and child health (CEMACH). Improving care for mothers, babies and children. Why mothers die 2000-2002. The sixth report of the confidential enquires into maternal deaths in the UK. Br J Obstet Gynaecol.

[25] Wildman K, Bouvier-Colle MH, Then MOMS group (2004). Maternal mortality as an indicator of obstetric care in Europe. Int J Obstet Gynaecol.

[26] National Institute for Health and Care Excellence (NICE). Intrapartum care for healthy women and babies. Clinical Guideline 2014 (CG190). http://www.nice.org.uk/guidance/CG190. Accessed 15 July 2018. 31820894

